# Hybrid resistance to parental bone marrow grafts in nonlethally irradiated mice

**DOI:** 10.1111/ajt.15146

**Published:** 2018-11-16

**Authors:** Benedikt Mahr, Nina Pilat, Nicolas Granofszky, Mario Wiletel, Moritz Muckenhuber, Svenja Maschke, Karin Hock, Thomas Wekerle

**Affiliations:** ^1^ Section of Transplantation Immunology Department of Surgery Medical University of Vienna Vienna Austria

**Keywords:** animal models: murine, basic (laboratory) research/science, bone marrow/hematopoietic stem cell transplantation, immunobiology, innate immunity, natural killer (NK) cells/NK receptors, rejection, tolerance: chimerism

## Abstract

Resistance to parental bone marrow (BM) grafts in F1 hybrid recipients is due to natural killer (NK) cell–mediated rejection triggered through “missing self” recognition. “Hybrid resistance” has usually been investigated in lethally irradiated F1 recipients in conjunction with pharmacological activation of NK cells. Here, we investigated BM‐directed NK‐cell alloreactivity in settings of reduced conditioning. Nonlethally irradiated (1‐3 Gy) or nonirradiated F1 (C57BL6 × BALB/c) recipient mice received titrated doses (5‐20 x 10^6^) of unseparated parental BALB/c BM without pharmacological NK cell activation. BM successfully engrafted in all mice and multilineage donor chimerism persisted long‐term (24 weeks), even in the absence of irradiation. Chimerism was associated with the rearrangement of the NK‐cell receptor repertoire suggestive of reduced reactivity to BALB/c. Chimerism levels were lower after transplantation with parental BALB/c than with syngeneic F1 BM, indicating partial NK‐mediated rejection of parental BM. Activation of NK cells with polyinosinic–polycytidylic acid sodium salt poly(I:C), reduced parental chimerism in nonirradiated BM recipients but did not prevent hematopoietic stem cell engraftment. In contrast, equal numbers of parental lymph node cells were completely rejected. Hence, hybrid resistance leads to incomplete rejection of parental BM under reduced conditioning settings.

## Abstract 2

Modifying conditioning in the hybrid resistance model reveals that lasting chimerism ensues after nonmyeloablative irradiation, even when no recipient irradiation is given, indicating that natural killer cell–mediated bone marrow rejection is incomplete in reduced conditioning settings.

**Abbreviations**
BM: bone marrow
GvHD: graft versus host disease
LN: lymph node
NK: natural killer
poly(I:C): polyinosinic–polycytidylic acid sodium salt
TBI: total body irradiation
TLR: toll‐like receptor

## 1 INTRODUCTION

Natural killer (NK) cells are large granular lymphocytes that serve as first‐line defense against pathogens and neoplastic cells.^1^ The expression of germline‐encoded receptors endows them to discriminate between healthy, infected, and malignant cells. It is also widely acknowledged that NK cells play a major role in the rejection of allogeneic bone marrow (BM), although they are not able to fully reject solid allografts.^2^, ^3^ The importance of NK cells in the rejection of allogeneic BM cells has been elegantly demonstrated when parental BM was transplanted into the first generation (F1) of offspring recipients (“hybrids”).^4^ Under these circumstances in which T cells exhibit no anti‐donor alloreactivity, the lack of recipient MHC molecules on donor cells triggers “missing‐self recognition” by recipient NK cells and leads to rejection of the parental BM (“hybrid resistance”).^5^, ^6^, ^7^ This model has ever since been a valuable tool for elucidating the role of NK cells in allogeneic BM transplantation but also has its limitations. In the classical hybrid resistance model, recipients are lethally irradiated and NK cells are actively preactivated with polyinosinic–polycytidylic acid sodium salt (poly(I:C)).^4^, ^8^ This model was developed in the early 1970s^6^ when myeloablative doses of irradiation were routinely used for BM transplantation. In recent decades, reduced conditioning regimens based on nonlethal irradiation have gained importance in the clinical setting of BM transplantation, but the effect of NK‐mediated hybrid resistance remains unclear under these conditions. To address this issue, we investigated hybrid resistance under reduced conditioning settings.

## 2 MATERIAL AND METHODS

### 2.1 Mice

CB6F1 (male C57BL/6 × female BALB/c; CD45.2) and BALB/c (CD45.2) mice were purchased from Charles River and congenic B6.SJL‐Ptprca Pepcb/BoyJ (CD45.1) mice from Jackson Laboratory. F1 (CD45.1/CD45.2) mice were obtained by crossing male CD45.1 on C57BL/6 background with female CD45.2 BALB/c mice. All mice were housed under specific pathogen‐free conditions and female mice were used between 8 and 12 weeks of age. All animal experiments were approved by the internal review board of the Medical University of Vienna and by the Austrian Ministry of Science and Research (permission number GZ: BMWFW‐66.009/0028‐WF/V/3b/2015).

### 2.2 Bone marrow transplantation

F1 (CD45.1/CD45.2) recipient mice received titrated doses (5‐20 × 10^6^) of unseparated BALB/c (CD45.2) or CB6F1 (CD45.2) BM cells (d0). Bones (femur, tibia, pelvis, and humerus) were flushed with a syringe and BM cells were collected in M199 medium (Sigma Aldrich) supplemented with 10 mM Hepes Buffer (MP Biomedicals) and 50 μg/ml gentamycin (MP Biomedicals).^9^ Indicated groups received varying doses (1‐3 Gy) of total body irradiation (TBI, d‐1) and selected BM recipients additionally received α‐NK1.1 (0.25 mg: d‐1, d2, d5, d8; clone PK136; BioXcell) or poly(I:C) (0.2 mg; d‐1; Sigma).

### 2.3 Secondary bone marrow transplantation

Primary recipients received 10 × 10^6^ BALB/c BM (d0) and poly(I:C) (0.2 mg, d‐1, Sigma). Sixteen weeks after transplantation, BM cells were recovered from primary recipients and transplanted into secondary F1 mice conditioned with 11 Gy TBI (2 × 5.5 Gy). On the day of reconstitution, each secondary recipient was transplanted with 20 × 10^6^ BM cells recovered from one chimera (i.v.).

### 2.4 Skin transplantation

Full‐thickness tail skin was grafted 4‐6 weeks after BM transplantation and visually inspected thereafter at short intervals. Grafts were considered to be rejected when less than 10% remained viable, as described earlier.^10^

### 2.5 Flow cytometry

The presence of donor cells was assessed at regular intervals by staining CD45.1 and CD45.2 on blood leukocytes. Donor chimerism was assessed as percentage of CD45.1^−^ CD45.2^+^ cells among CD45.1^+^ CD45.2^+^ plus CD45.1^−^ CD45.2^+^ leukocytes (CD45.1^−^ CD45.2^+^/(CD45.1^−^ CD45.2^+^ + CD45.1^+^ CD45.2^+^) × 100). APC anti‐mouse CD45.1 (A20), PE anti‐mouse CD45.2 (104), FITC anti‐mouse Mac‐1 (M1/70), PE‐Cy7 anti‐mouse CD8 (53‐6.7), APC‐Cy7 anti‐mouse CD4 (RM4‐5), Pacific Blue anti‐mouse CD3 (17A2), FITC anti‐mouse CD49b (DX5), FITC anti‐mouse NK1.1 (PK136), PE anti‐mouse Ly49D (4E5), biotin anti‐mouse Ly49A (YE1/48.10.6) were purchased from Bio Legend. PE‐Cy7 anti‐mouse Ly49G2 (4D11) was purchased from eBioscience.

### 2.6 Statistical analysis

Data were statistically analyzed with GraphPad Prism 5.0 (Graph Pad Inc., La Jolla, CA). A 2‐sided Student's *t* test with equal variances was used to compare chimerism levels. Total chimerism levels were compared between groups by using analysis of variance (ANOVA). The correlation between BM dose and chimerism level was assessed by a linear regression model. A *P*‐value below .05 was considered to denote statistical significance (*P < .05, **P < .01, ***P < .001, ****P < .0001, n.s. *P* > .05).

## 3 RESULTS

**Figure 1:**
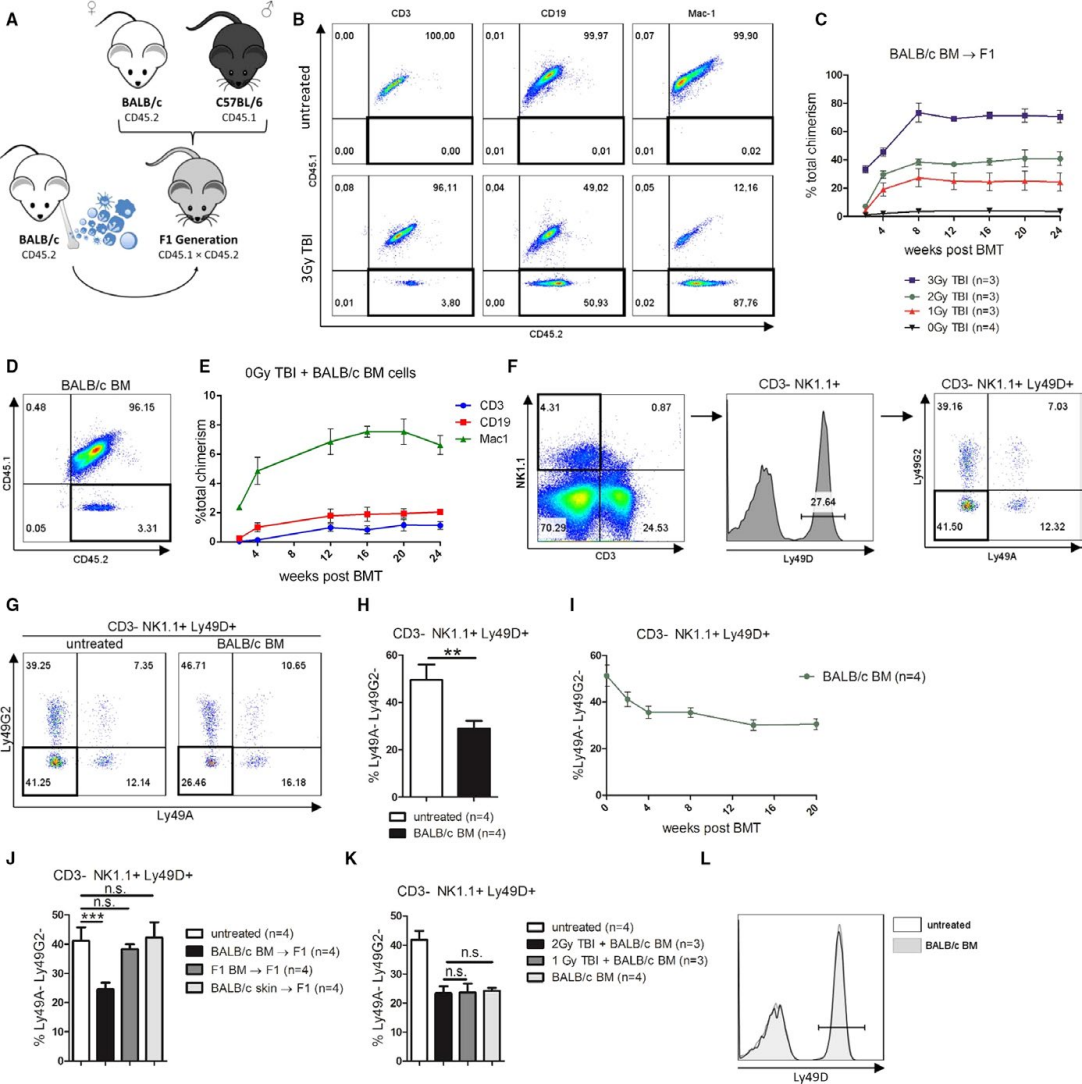
Hybrid resistance in recipients treated with nonlethal or no irradiation. (A) Schematic illustration of the hybrid resistance model allowing long‐term tracking of chimerism. (B) F1 recipients received 3 Gy TBI and 20 × 10^6^ BALB/c BM cells or remained untreated. Recipient leukocytes coexpress CD45.1 and CD45.2, whereas donor cells solely express CD45.2 (lower right quadrant). (C) F1 recipients of BALB/c BM received titrated doses of TBI. Mean percentages ± standard deviation (SD) of total donor chimerism (ie, CD45.2+  CD45.1‐ cells among CD45.2+ leukocytes) in blood was measured by flow cytometry and is shown over time. (D, E) Nonirradiated F1 mice received 20 × 10^6^ BALB/c BM cells. (D) Dot plot shows donor cells (CD45.2+ CD45.1−) in the BM 24 weeks posttransplant. Representative mouse is shown. (E) Mean percentages ± SD (n = 4) of donor chimerism among specific lineages is shown over time. (F) Gating strategy to identify CD3− NK1.1+  NK cells, which express the activating receptor Ly49D but none of the inhibitory receptors Ly49A or Ly49G2. (G, H) NK cell receptors were analyzed in the spleen of untreated F1 mice or F1 recipients of BALB/c BM 24 weeks posttransplant. (G) Dot plot illustrates reduction of Ly49D+ Ly49A/G2‐ NK cells in F1 mice receiving BALB/c BM. Representative mice are shown. (H) Bars represent mean ± SD of splenic Ly49D+ Ly49A/G2‐ NK cells. (I) Nonirradiated F1 recipients received BALB/c BM and NK cell receptors were analyzed at regular intervals in the blood. Mean percentages ± SD of Ly49A/G2‐ Ly49D+ NK cells are shown over time. (J‐L) Splenic NK cell receptors were analyzed in F1 mice receiving indicated treatments in the spleen 24 weeks after BM transplantation. (J) Selected groups of F1 mice received BM or skin grafts from indicated donors. Bars depict mean percentages ± SD of Ly49A/G2‐ Ly49D+ NK cells. (K) F1 mice received indicated doses of TBI and 20 × 10^6^ BALB/c BM. Bars represent mean percentages ± SD of splenic NK cells expressing Ly49D without Ly49A/G2. (L) Ly49D expression on splenic NK cells was compared between F1 mice transplanted with or without BALB/c BM. Histogram overlay shows Ly49D expression on NK cells. Representative mice are shown [Color figure can be viewed at wileyonlinelibrary.com]

To track BM engraftment and chimerism for an extended period, we crossed C57BL/6 (CD45.1) male mice with BALB/c (CD45.2) female mice so that the resulting F1 generation coexpressed both CD45.1 and CD45.2, whereas donor BALB/c cells solely expressed CD45.2 (Figure 1A). F1 recipient mice were irradiated (d‐1) with 3 Gy TBI and received 20 × 10^6^ BALB/c BM (d0). All recipients developed high levels of persistent multi‐lineage mixed chimerism (≈70% total leukocyte chimerism) (Figure 1B and C). With a reduced dose of irradiation of 2 and 1 Gy, the ensuing levels of total donor chimerism declined but stable chimerism was still induced (Figure 1C). Even in the absence of any irradiation, multi‐lineage chimerism was detectable in all mice and persisted long‐term in blood (Figure 1C‐E) and BM (follow‐up 24 weeks), implying successful stem cell engraftment. To discern whether NK cells in stable mixed chimeras would adapt to the chronic exposure of donor cells we analyzed a subset of NK cells expressing the activating receptor Ly49D, which binds the BALB/c specific MHC class I molecule H2D^d^.^11^, ^12^ Ly49D^+^ NK cells can simultaneously express inhibitory receptors (Ly49A, Ly49G2) that bind the very same MHC molecule.^13^ Those NK cells that express the activating receptor Ly49D without expressing any of the inhibitory receptors Ly49A or Ly49G2 are potentially donor‐reactive^9^, ^14^ (Figure 1F). Transplantation of BALB/c BM into nonirradiated F1 mice significantly reduced the amount of Ly49D^+^ Ly49A/G2^−^ NK cells (Figure 1G, H). The rearrangement of the NK cell receptor repertoire evolved over the first 4 weeks posttransplant and remained stable thereafter (Figure 1I). NK cell adaption did not occur if allogeneic skin or syngeneic BM was transplanted (Figure 1J). The degree of NK cell receptor rearrangement was independent of the dose of irradiation and the ensuing levels of chimerism (Figure 1K). Transplantation of parental BM altered the appearance of inhibitory receptors but had no effect on the expression of the activating receptor Ly49D (Figure 1L). Thus, in recipients receiving no or nonlethal irradiation, NK cells did not abrogate engraftment of parental BM, but rather adapted through the rearrangement of their receptor repertoire.^15^

**Figure 2:**
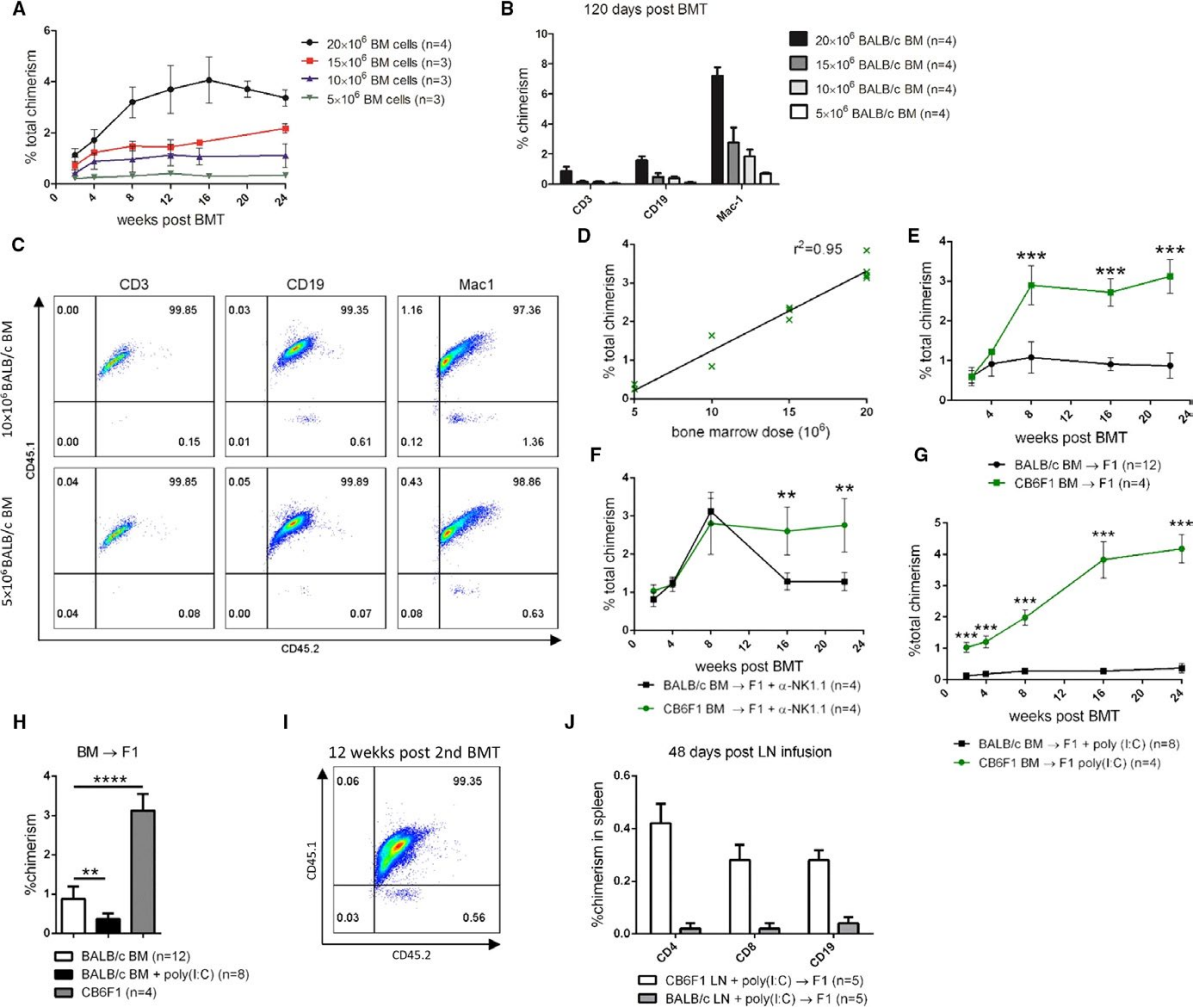
NK cells only partially reject parental BM cells in nonirradiated recipients. (A) Nonirradiated F1 recipients received indicated doses of BALB/c BM cells. Mean percentage of total leukocyte chimerism (ie, CD45.2 +  CD45.1‐ cells among CD45.2 +  leukocytes) ± SD in the blood is shown over time. (B, C) Nonirradiated F1 recipients received indicated doses of BALB/c BM. (B) Mean percentages of donor chimerism ± SD of distinct leukocyte populations in blood is shown 24 weeks after BM transplantation. (C) Dot plot shows donor chimerism among CD3+, CD19+, and Mac1+ cells 24 weeks after BM transplantation for indicated BM doses. (D) Correlation between donor leukocyte chimerism and transplanted BM dose 24 weeks after BM transplantation. (E‐G) Nonirradiated F1 recipients received either 10 × 10^6^ BALB/c or 10 × 10^6^ F1 BM cells. Mean percentage ± SD of total donor chimerism is shown over time. Indicated groups received α‐NK1.1 or poly(I:C). (H) Mean percentages of donor chimerism (±SD) in the blood is compared between nonirradiated recipients of BALB/c BM treated with or without poly(I:C) as well as recipients of F1 BM at the end of the follow‐up. (I) BM was recovered from poly(I:C)‐treated F1 chimeras 12 weeks after BM transplantation (n = 3) and transplanted into lethally irradiated secondary F1 recipients. Donor chimerism was measured in the blood 12 weeks after the secondary BM transplantation. Representative dot plot is shown. (J) Bars illustrate mean chimerism ± SD of indicated leukocyte populations 7 days postinfusion of parental lymph node cells into nonirradiated F1 recipients [Color figure can be viewed at wileyonlinelibrary.com]

Next, we decreased the numbers of BALB/c BM cells transplanted into nonirradiated F1 mice. Donor leukocyte chimerism was detectable long‐term with all applied BM doses, even with the lowest dose of 5 × 10^6^ cells (Figure 2A). Multi‐lineage chimerism, however, developed only with a BM dose of 10 × 10^6^ or higher, as no CD3 or CD19 chimerism was detectable with 5 × 10^6^ cells (Figure 2B, C). Overall, the dose of transplanted BM cells and the level of leukocyte chimerism showed a linear correlation (Figure 2D). To determine whether partial rejection of parental BM cells occurs in successful chimeras, we compared chimerism levels between recipients of parental (BALB/c) and syngeneic (CB6F1) BM. Chimerism levels were significantly lower from 8 weeks posttransplant on in recipients of parental compared to recipients of syngeneic BM (Figure 2E). Temporarily depleting NK cells (α‐NK1.1) at the time of transplantation transiently equalized chimerism levels between both groups (Figure 2F), although parental chimerism again declined 8 weeks posttransplant at the time when NK cells slowly recur.^9^ In the absence of irradiation, preactivating NK cells with the toll‐like receptor (TLR)‐3 agonist poly(I:C) 1 day prior to transplantation significantly reduced parental chimerism, but did not lead to complete loss of chimerism with distinct populations of donor cells persisting long‐term in different tissues (Figure 2G, H). To assess directly whether hematopoietic stem cells had successfully engrafted despite poly(I:C) treatment, BM was recovered from chimeras 12 weeks posttransplant and was transplanted into lethally irradiated secondary F1 recipients. Multi‐lineage chimerism was detectable in secondary recipients, demonstrating that donor hematopoietic stem cells had indeed engrafted and survived in primary recipients^16^, ^17^ (Figure 2I). To test whether NK cells would be more potent in rejecting types of donor hematopoietic cells other than BM, we injected 10 × 10^6^ BALB/c lymph node cells into F1 recipients. Donor lymphocytes were completely rejected (<0.05% chimerism) 7 days postinfusion by F1 recipient (Figure 2J).

## 4 DISCUSSION

The results presented herein reveal that under reduced conditioning settings, parental BM is only partially rejected by NK cells in F1 recipients. Multi‐lineage chimerism ensues even in nonirradiated F1 recipients transplanted with moderate BM doses. Complete rejection of parental BM is not triggered, even when poly(I:C) is given.

Irradiation promotes engraftment by creating space in the BM niche,^18^ but also leads to the release of proinflammatory cytokines^19^, ^20^ and other danger signals that are expected to enhance NK alloreactivity.^1^, ^21^ Therefore it is tempting to speculate that NK cell alloreactivity was mitigated under reduced intensity conditioning. Pharmacological NK stimulation with poly(I:C) led to complete rejection of parental lymphocytes but not parental BM, allowing stem cell engraftment in this setting. The reasons why NK cells preferentially target lymphohematopoietic cells remain unclear but likely reflect the selective expression of distinct receptors.^22^ It has also been suggested that NK cells exhibit no direct cytotoxicity against stem cells^17^, ^23^ and that they reside within immune privileged sites that prevent them from undesired immune attack.^24^

So far the proliferation of recipient splenocytes shortly after BM transplantation served as surrogate marker for BM engraftment in hybrid resistance models using lethally irradiated mice.^4^ This end point, however, does not allow drawing robust conclusions regarding long‐term chimerism.^25^ The model presented herein provides a new oppurtunity to follow parental donor cells in F1 recipients by flow cytometry and is thus particularly suited for the investigation of NK‐mediated hybrid resistance under distinct reduced conditioning settings. Our results demonstrate that NK‐mediated rejection of parental BM is diminished and remains incomplete in nonlethally irradiated recipients. Long‐term multi‐lineage chimerism was observed even in nonirradiated recipients. This finding also indicates that “space” does not necessarily have to be created in the recipient through myelosuppression for hematopoietic stem cells to engraft. This has already been suggested previously in models of high‐dose BM administration,^26^, ^27^ and recently it has been reported that ample free sinusoidal perivascular niches exist where exogenous stem cells can engraft.^16^

Even if allogeneic stem cells have sufficient space to engraft, one would expect NK cells to resist their engraftment unless very large BM doses are infused.^15^ Unexpectedly, NK adaptation occurred at moderate BM doses, reminiscent of the NK adaptation seen with the chronic exposure of viruses that is associated with decreased expression of the activating receptor Ly49H.^28^ However, we did not observe alterations in the expression of the donor‐specific activating receptor Ly49D in established mixed chimeras. It rather seemed that Ly49D^+^ NK cells would obtain the expression of the inhibitory receptors Ly49A and/or Ly49G2. This adaptation extended over a period of 4 weeks, which approximately corresponds to the time of NK cell maturation in the BM.^29^ The altered expression of Ly49 inhibitory receptors in MHC class I deficient mice and in fully allogeneic BM chimeras further supports this assumption.^14^, ^30^

Our data from the murine hybrid resistance setting suggest that NK‐mediated BM rejection is less potent in reduced conditioning settings than in lethal irradiation regimens, allowing stem cell engraftment with moderate BM doses even in nonirradiated recipients.

## DISCLOSURE

The authors of this manuscript have no conflicts of interest to disclose as described by the *American Journal of Transplantation*.
